# Impact of Elective Courses and Gender Influence on Enhancing Radiological Anatomy Understanding Among Medical Students: A Cross-Sectional Study

**DOI:** 10.7759/cureus.101354

**Published:** 2026-01-12

**Authors:** Abdulaziz H Moria, Mohammed T Hakami, Talal F Almaghnam, Nasser M Alnasib, Ayat J Aleid, Fatimah A Al Mubarak, Jawri A Al Amri, Mohammed Bahgat

**Affiliations:** 1 Biomedical Sciences, King Faisal University, Al-Ahsa, SAU; 2 College of Medicine, King Faisal University, Al-Ahsa, SAU

**Keywords:** al-ahsa, anatomy, cross-sectional study, education, elective course, king faisal university, medical school, radiological anatomy, radiology

## Abstract

Rationale and objectives

Gross anatomy remains a pivotal part of medical education. Specially, when integrating with radiology. Radiological education and teaching modalities improved in efficacy with earlier exposure, providing beneficial outcomes regarding diagnostic expertise. Studies demonstrate radiological images significantly enhance students’ applied knowledge in clinical settings, along with introducing problem-based and self-directed learning.

Materials and methods

At King Faisal University (KFU), College of Medicine, in Al Hofuf, Saudi Arabia, a cross-sectional study was established to assess radiological anatomy understanding of third-year medical students, with a population size of 263 (55.1%, n = 145) males and (44.9%, n = 118) females. The collected data include demographics, academic performance, and elective course attendance. The study includes all third-year medical students, males and females, who have and have not taken the elective course. The study excludes male and female students of all other academic years. The data were analyzed by using IBM SPSS Statistics for Windows, version 29.

Results

The current study of 199 third-year students included 80 females (40.2%) and 119 males (59.8%). The mean age was 21.603 (±0.649), with n = 84 aged ≤21 (42.2%) and n = 115 aged >21 (57.8%). The mean understanding score for elective course non-attendees was 4.55/20 (standard deviation (SD) = 3.1), while for course attendees, it was 14.17/20 (SD = 3.9), which is significantly higher (p < 0.001). Females demonstrated a significantly higher understanding (48.75%, n = 39) compared to males (32.8%, n = 39) (p = 0.019). Elective course attendees showed significantly higher understanding (97.9%, n = 47) in comparison to non-attendees (21.2%, n = 32) (p < 0.001). Gender (p = 0.033) significantly influenced high-level understanding, with males less likely (Exp(B) = 0.403, 95% CI (0.175, 0.930)).

Conclusion

The present study highlights the significant positive impact of elective courses on radiological anatomy understanding. Female participants and elective course attendance positively enhanced high-level comprehension, emphasizing educational interventions' effectiveness.

## Introduction

Gross anatomy remains an essential and fundamental component of medical education, encompassing both classroom lectures and practical sessions [1]. Problem-based and self-directed learning has become more popular than in previous generations due to its positive impact on students' skills in clinical settings and to adjustments in delivered volume and method [2]. In many preclinical anatomy courses, radiology education serves a significant role. It is commonly accepted that radiological imaging is an essential part of clinical diagnosis and patient management [3]. Students can discover the clinical significance of the anatomy they have learned from images, models, and body parts provided during lectures and practical sessions. Radiographs and imaging software programs were used to assist students in achieving a deeper comprehension of radiology and topographical anatomy [4]. In addition to radiology, we must promote state-of-the-art clinical knowledge, further development of skills, early practical experience, evaluation of competence and professionalism, and prepare students to deliver healthcare in a world with constantly evolving technologies and economic limitations [5].

As previous studies reported, the efficacy of anatomical teaching in radiology programs is high [6]. A study published in 2016 stated that the ability to choose a radiological modality improves with clinical practice, yet there is still an opportunity for development to satisfy the requirements of a general radiology elective. Second-year students showed the most progress in this subject, indicating that starting radiology education earlier is beneficial [7]. In 2022, a study revealed that over 80% of students claimed that radiological images enhanced their applied knowledge in clinical settings and assisted them in meeting their educational goals. According to this study's findings, teaching anatomy through radiographic images has a significantly elevated positive rate among students [8]. Previous studies have been conducted in various medical schools across different regions, such as the Johns Hopkins School of Medicine radiology elective, Gulf Medical University, Sungkyunkwan University School of Medicine, Pritzker School of Medicine, and the University of Chicago [4-7]. King Faisal University (KFU) conducts radiological anatomy as an elective course for third-year students. The course started with an introduction to discuss the history of radiology, its significance, X-ray interpretation, different radiographic densities, various radiographic modalities, and fundamental principles for interpreting various radiographs (normal and abnormal conditions). Although previous studies demonstrate that radiological images significantly enhance applied knowledge in clinical settings [1-9], no previous similar study has been conducted at KFU, Saudi Arabia. According to this, the current study was designed to emphasize the impact of radiological anatomy courses on students’ clinical sense.

Objectives 

The current study aims to evaluate the impact of the radiological anatomy elective course on third-year medical students’ skills of identifying normal anatomical structures and abnormal findings in different radiographic modalities and comparing the results between the students who attended and those who did not attend the course. The authors hypothesize that the students who attended the radiological anatomy elective course will demonstrate a significantly higher ability to interpret various imaging modalities and accurately identify the normal and abnormal appearance of different anatomical structures, compared to students who did not attend the course.

## Materials and methods

A cross-sectional study was conducted among medical students from the College of Medicine at KFU in Al Ahsa, Saudi Arabia. An eight-week radiological anatomy elective course was offered to 57 third-year medical students, with n = 30 males (52.6%) and n = 27 females (47.4%); however, only 48 students participated in the study. The radiological anatomy course enables students to identify the anatomical structures in different radiographic varieties. The course consisted of four labs and 11 lectures. The four labs (thorax, abdomen, upper limb, and lower limb) were designed to describe specific gross anatomy and sectional anatomy information, which enabled the students to identify anatomical structures in different radiographic modalities. Each lab is followed by a number of lectures related to each region, for the identification of related anatomical structures in plain X-ray, CT, and MRI radiographs of normal and diseased patients.

A systematic process was followed to develop a quiz-based questionnaire that was validated by anatomy and radiology experts. The first section was prepared to gather personal demographic information, including age, gender, academic year, GPA score, and whether the student has attended the course or not. The second section consisted of 20 radiograph-based questions.

Our target population included all third-year medical students, whether registered for the course or not. The total number of third-year medical students was 263, with n = 145 males (55.1%) and n = 118 females (44.9%). The estimated minimum sample size is 157. Given a 95% confidence level, the margin of error was 5% (5% absolute precision), and the response distribution was 50%. The Rao soft calculator was used to compute the sample size. The total number of participants is 199 students, with n = 119 males (59.8%) and n = 80 females (40.2%). The study included all third-year medical students, males and females, who had and had not taken the elective course. The study excluded male and female students of all other academic years.

The questionnaire was distributed to the participants and was expected to be completed within seven to 10 minutes. In this study, we took written consent from all participants. They were informed of the study's objective, how the data are used, the benefits and risks involved, and how the data are confidential and anonymous. The participants were not offered incentives to participate in the study.

The reliability analysis yielded a Cronbach's alpha of 0.894, indicating good internal consistency. All items had corrected item-total correlations above 0.3, and removing any item did not significantly increase the overall alpha value.

Ethical approval and consent to participate

The study was approved by the KFU Deanship of Scientific Research's Ethical Committee (ETHICS2016). It was optional to take part in the study. The study's objective was well-defined, and an anticipated time frame was provided. The data collected throughout the survey were kept confidential, and the participants used codes to symbolize their anonymous responses.

Data processing and analysis 

The data were collected and analyzed using the IBM SPSS Statistics for Windows, version 29.0 (released 2022, IBM Corp., Armonk, NY). The descriptive analysis was done to show mean, median, and mode values with standard deviation (SD) for quantitative data. The categorical data were analyzed, and the chi-square was used to assess any association among different variables. The effect of a single variable on other variables was assessed through logistic regression analysis.

Statistical analysis

An extensive and thorough statistical analysis was conducted on the data, embracing both descriptive and inferential methodologies. Initially, a descriptive analysis was conducted to summarize the demographic characteristics of the participants, which included age, gender, academic year, and performance, attended the elective course for radiological anatomy or not. To assess the awareness and knowledge score difference between variables, inferential analyses such as the Mann-Whitney U T-test (for two groups) were utilized. To see the association, Chi-square and Fisher’s exact were used, and to see the predictor of high-level knowledge, binary logistic regression was employed. A p-value of 0.05 or lower and a 95% confidence interval are required for statistical significance to be established. All statistical analyses were conducted using IBM SPSS Statistics for Windows, version 29.0 (released 2022, IBM Corp., Armonk, NY).

## Results

A total of 199 students, all of whom were third-year medical students, were included in our study. The preponderance of the study sample was comprised of males (59.8%, n = 119). In regard to age demographics, a significant majority of participants (57.8%, n = 115) were more than 21 years of age, in comparison to n = 84 (42.2%) who were aged 21 or younger (mean = 21.603 ± 0.649). Academic performance varied, with n = 74 (37.2%) participants achieving a grade point average (GPA) between 4.75 and 5.0, while in contrast, 9.0% (n = 18) of the participants achieved a grade of less than 4.0. For the radiological anatomy elective course attendance, n = 48 (24.1%) had attended, while n = 151 (75.9%) participants had not attended (Table 1).

**Table 1:** Sociodemographic and other parameters of the participants

		Frequency (n = 199)	Percent
Gender	Female	80	40.2%
	Male	119	59.8%
Age	≤21 years	84	42.2%
	>21 years	115	57.8%
Batch year	3rd Year	199	100.0%
Academic performance	< 4.0	18	9.0%
	4.0 to <4.5	47	23.6%
	4.5 to <4.75	60	30.2%
	4.75-5.0	74	37.2%
Attendance of the Radiological Anatomy course	Yes	48	24.1%
	No	151	75.9%

**Figure 1:**
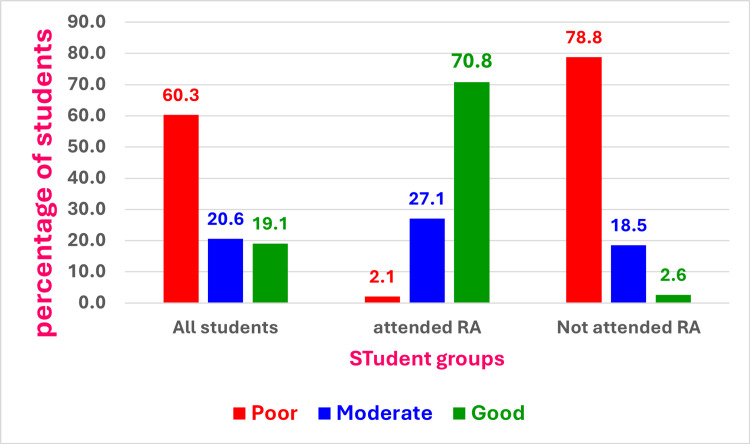
Knowledge level of students presented as all students, students attended Radiological anatomy (RA) course and students did not attend RA course. The performance of the students attended the course was much better than students who did not attend the course. Image created by the authors with IBM SPSS Statistics version 29.0

Among all the students (N = 199) who were assessed within the domains of radiological anatomy, 60.3% (n = 120) demonstrated poor knowledge, 20.6% (n = 41) exhibited a moderate level of knowledge, and 19.1% (n = 38) participants displayed good knowledge. Thirty-four (70.8%) participants who attended the course displayed good knowledge, contrasting with four non-attendees (2.6%). On the other hand, 78.8% (n = 119) of the participants who did not attend the elective course demonstrated poor knowledge, in comparison to only 2.1% (n = 1) who had attended the RA course (Figure 1).

**Figure 2:**
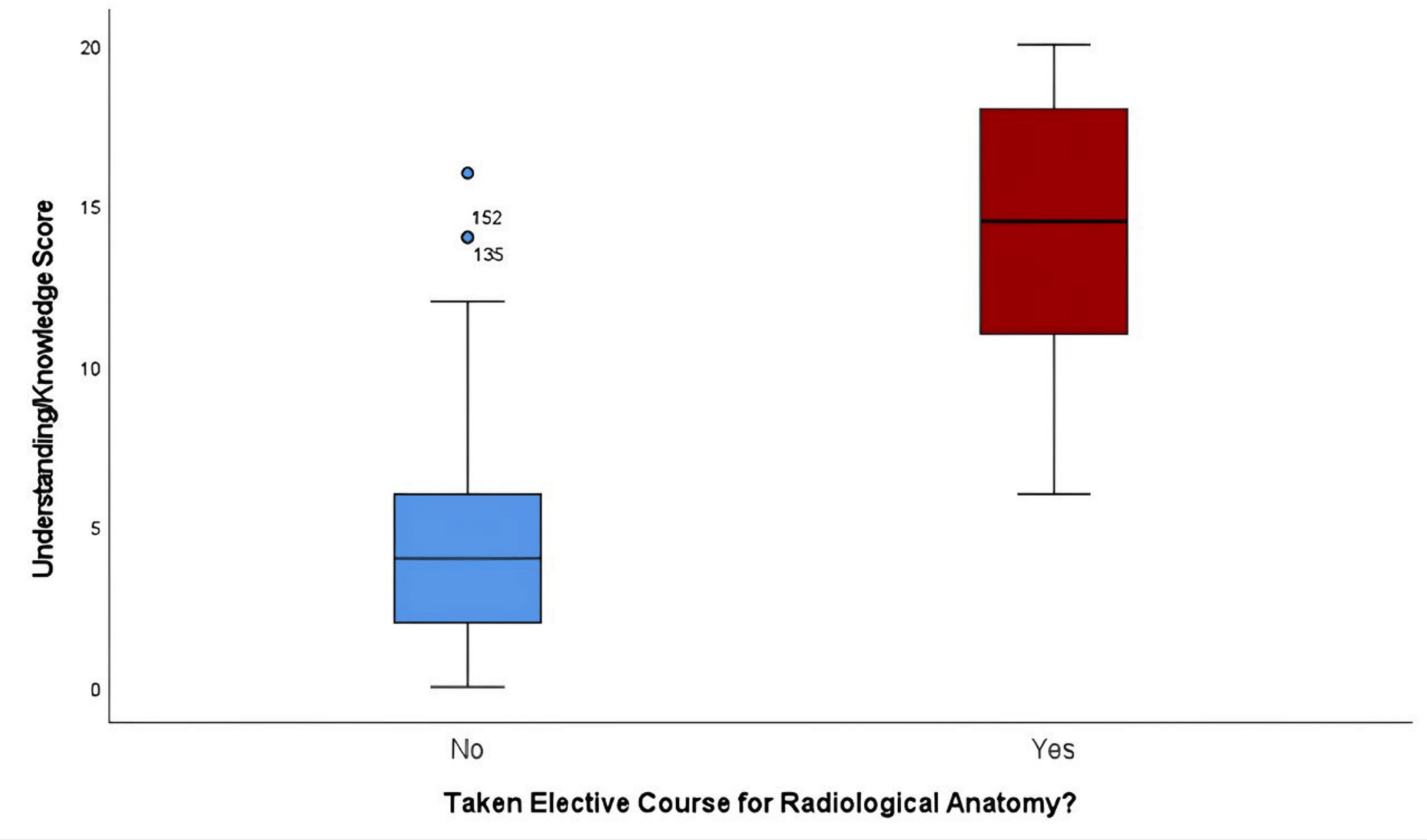
Box plot showing the difference in knowledge score based on the elective course taken or not. The knowledge score was much higher in students who attended the course. Image created by the authors with IBM SPSS Statistics version 29.0

Table 2 and Figure 2 show the effectiveness of the elective course in enhancing the understanding of radiological anatomy among participants. The mean understanding score for all the participants was found to be 6.87/20 (SD = 5.3). Regarding the participants who did not attend the elective course (75.9%, n = 151), the mean understanding score was 4.55/20 (SD = 3.1); on the other hand, for those who did attend the course (24.1%, n = 48), the mean score greatly increased to 14.17/20 (SD = 3.9). Using the Mann-Whitney U test, a significant difference in scores was discovered between the two participant groups (U = 236.500, Z = -9.779, p < 0.001), indicating that the elective course had a beneficial effect on participants' comprehension of radiological anatomy concepts.

**Table 2:** Effectives of elective course in understanding of radiological anatomy.

		N	Mean (SD)	Mann-Whitney U	Z	Sig. value
Attendance of the Radiological Anatomy course	Yes	48	14.17/20 (3.9)	236.500	-9.779	<0.001
	No	151	4.55/20 (3.1)			
	Total	199	6.87/20 (5.3)			

Table 3 illustrates how the elective course has improved knowledge of radiological anatomy and how it varies across different domains. Gender differences were found to be statistically significant (p = 0.019), with a greater proportion of females (48.75%, n = 39) demonstrating higher knowledge than males (32.8%, n = 39). Age, on the other hand, did not significantly alter knowledge levels (p = 0.284). Similarly, there was no significant difference in GPA values (p = 0.239) among the various understanding levels. Participants who attended the elective course demonstrated significantly higher knowledge levels than those who did not (p < 0.001). The difference was notable: only 21.2% (n = 32) of non-attendees had excellent knowledge levels, compared to 97.9% (n = 47) of course attendees.

**Table 3:** Effect of gender, age, GPA, and taking elective course on the understanding of radiological anatomy.

			Knowledge level about radiological anatomy		Sig. value
			Low level (<50th percentile)	High level (>50th percentile)	
Gender	Female	N	41	39	0.019
		%	51.25%	48.75%	
	Male	N	80	39	
		%	67.2%	32.8%	
Age	≤ 21 Years	N	47	37	0.284
		%	56.0%	44.0%	
	˃ 21 Years	N	73	42	
		%	63.5%	36.5%	
GPA score	< 4.0	N	11	7	0.239
		%	61.1%	38.9%	
	4.0 to <4.5	N	34	13	
		%	72.3%	27.7%	
	4.5 to <4.75	N	32	28	
		%	53.3%	46.7%	
	4.75-5.0	N	43	31	
		%	58.1%	41.9%	
Attendance of the Radiological Anatomy course	No	N	119	32	<0.001
		%	78.8%	21.2%	
	Yes	N	1	47	
		%	2.1%	97.9%	

Table 4 shows a high level of understanding of radiological anatomy derived from multiple aspects during the analysis. Age was not significantly correlated with other characteristics in our data after being adjusted for them (p = 0.616, Exp(B) = 1.239, 95% CI (0.536, 2.862)). However, gender shows a significant impact (p = 0.033), with males being less probably to achieve high-level understanding compared to females (Exp(B) = 0.403, 95% CI (0.175, 0.930)). GPA scores showed some trends (p = 0.271); it was clear that students with higher GPAs achieved higher understanding (Exp(B) = 1.276, 95% CI (0.827, 1.969)). Taking elective courses for radiological anatomy was the most significant predictor (p < 0.001); it increased the likelihood of high-level understanding (Exp(B) = 201.481, 95% CI (25.933, 1565.393)). The constant term was not significant (p = 0.506). The importance of the elective course in predicting high-level understanding of radiological anatomy was clear from these highlighted results, even when adjusting for other factors.

**Table 4:** Adjusted predictors of high-level understanding about radiological anatomy (multivariate analysis).

	B	Sig.	Exp(B)	95% CI	
				Lower	Upper
Age	.0214	0.616	1.239	0.536	2.862
Gender (male)	-0.909	0.033	0.403	0.175	0.930
GPA score	0.244	0.271	1.276	0.827	1.969
Taken elective courses for Radiological Anatomy? (Yes)	5.306	0.000	201.481	25.933	1565.393
Constant	-6.185	0.506	0.002		

## Discussion

Gross anatomy remains central in medical education, as does the integration of problem-based and self-directed learning in radiology education to improve and enhance clinical relevance and expertise. Our study revealed the importance of the radiological anatomy elective course in improving the knowledge and skills of the students in identifying anatomical structures in different radiographic modalities. This confirms the results of previous research related to radiology. Sbayeh et al. (2016) asserted a close relationship between anatomy education and the interpretation of diagnostic imaging scans [8]. High efficacy is demonstrated by radiological education, with earlier exposure yielding more favorable and advantageous results. Students' perceptions of radiology as a specialty and interest in radiology as a career are improved, according to Branstetter et al. (2007), when they are exposed to radiology during their first year of medical school [9]. According to earlier studies, applying radiological images in clinical settings significantly enhances applied knowledge. Students believed it was beneficial to have radiological photos introduced during anatomy classes [6].

Our research provided important new information about the effects of these instructional interventions on students' knowledge levels, the demographic variables that affect comprehension, and the characteristics that indicate high-level comprehension. A major finding was the significant improvement in knowledge, competence, and skills reported by students registered in the elective course on radiological anatomy. Those who attended the course had a mean score that was markedly higher than those who did not. This indicates the value of focused educational interventions in facilitating students’ comprehension of complex anatomic concepts, especially in relation to radiology imaging procedures. According to Rathan et al. (2022), such interventions drive interest in radiology as a career path and promote radiological skills to comprehend demanding anatomical concepts [6].

Our results align with the broad consensus that elective-based interventions drive interest and promote the skills necessary to comprehend demanding anatomical concepts. However, a detailed comparison with existing literature reveals important methodological distinctions. For instance, Leschied et al. (2013) utilized a prospective pre- and post-test design with a control group to measure 3-day knowledge gains in imaging appropriateness among second-year students. In contrast, our study employs a cross-sectional approach with a larger cohort (N=199), evaluating the impact of an 8-week course specifically focused on regional and sectional anatomy. This suggests that the benefits of targeted instruction are not limited to short-term clinical appropriateness but extend to a deeper topographical understanding of human structures [10]. Good elective program experiences can also improve radiologists' enthusiasm and passion toward teaching, as well as students' comprehension of radiography and attitude toward the field [11]. Furthermore, previous studies have shown that attending elective courses in radiological anatomy improves students' comprehension of radiographic images and anatomical landmarks, which promotes their capacity in diagnosing patients, as well as making clinical decisions. In a comparable manner, a previous study revealed that students in their third and sixth years of study have a preference for elective courses that enable them to further develop their understanding, competency, and expertise in clinical radiology [12].

The significant improvement in knowledge observed in our elective course attendees mirrors a broader recognition in the literature regarding anatomical deficiencies. For instance, a recent study by Sarangi et al. (2024) among post-graduate radiology residents in India revealed that nearly 98% face challenges in image interpretation due to anatomical gaps, with a vast majority (91.5%) believing that short, human anatomy postings would significantly improve their radiological reporting skills. This underscores that the integration of radiology and anatomy is not merely a preclinical requirement but a continuous necessity. The need for such integration is further highlighted by the fact that (59%) of residents report a lack of confidence in the anatomical knowledge required for radiodiagnosis. To address these gaps, there is an overwhelming consensus (90%) among trainees for the implementation of a standardized human anatomy curriculum within specialty training. Furthermore, evidence suggests that a symbiotic approach, such as mentorship programs led by either experienced radiologists or anatomists, is highly favored by 88.5% of residents to bridge these knowledge gaps. This integrated model is further supported by the interest of 95% of residents in leveraging advanced technological tools, such as 3D reconstruction and virtual reality (VR) simulations, which can enhance human anatomy learning for radiologists [13].

Notably, in this study, we explored the impact of participants' academic background, age, and gender on their understanding of radiological anatomy. Gender emerged as a significant predictor, with females more likely to achieve high levels of understanding compared to males. This finding fits with other research that suggests differences between men and women in their ability to interpret radiological images. Similarly, Leon et al. (2022) show that female radiologists tend to have a narrower scope of practice and make fewer mistakes due to more knowledge than their male counterparts, even after detailed adjustment for factors that might explain gender differences in scope of practice and error [14].

Age did not significantly affect comprehension levels. However, the GPA score suggested a trend towards higher understanding as academic performance increased, indicating that students with stronger academic backgrounds frequently exhibit greater benefits from elective courses in radiological anatomy. Similarly, Brashi et al. (2024) and Mann et al. (2023) show a significant correlation between high levels of knowledge and students with the highest GPA (p ≤ 0.05) [15,16].

Hence, the results we obtained reflect the positive effects of the offered elective courses on students' understanding of radiological anatomy when compared with prior studies. However, by offering thorough insights into the demographic variables and determinants connected to high-level comprehension in this field, this study offers a distinctive contribution to the scope of existing literature. Previous studies have predominantly evaluated the effectiveness of educational interventions. However, our investigation delves deeper into the intricate relationships between demographic variables and knowledge acquisition, contributing to a better understanding of the multifaceted variables influencing medical education outcomes [2,3,5,10,11].

Limitations and future directions

Despite the valuable findings of this study, some limitations should be considered. First, as this research was a cross-sectional study, the findings lack causality. Second, the study’s confinement to a single institution limits the generalizability of the findings. Prospective studies are recommended to evaluate the long-term effect of the elective course in radiological anatomy. Future research should employ a multi-center design with a larger sample size to enhance the validity of the findings. Future researches on the integration of AI-based imaging tools, blended learning environments, and longitudinal tracking of clinical importance are required to assess retained knowledge.

## Conclusions

The findings of this study highlight the significant role of elective courses in improving medical students' knowledge of radiological anatomy. Students who participated in these specialized courses demonstrated a marked improvement in their knowledge and skills compared to their peers who did not take the elective. In addition, demographic variables such as gender were found to affect the results. This suggests that medical education programs should integrate targeted educational interventions to enhance students' radiological anatomy proficiency. Further research is needed to explore innovative teaching strategies and assess their effectiveness in preparing medical students to meet future clinical demands. Future research on the integration of AI-based imaging tools, blended learning environments, and longitudinal tracking of clinical importance is required to assess retained knowledge.
